# Foreign Medical and Physical Science and Literature

**Published:** 1819-04

**Authors:** 


### FOREIGN MEDICAL AND PHYSICAL SCIENCE AND LITERATURE.

### *Exposition of the Doctrine of* M. Broussais.

(*Continued from p.* 251.)

A STUDIOUS investigation of the causes, symptoms, and terminations, of the diseases which have formed the subject of our reflections; the results obtained from different modes of treatment; and particularly the examination of the bodies of those who fell victims to the violence of their maladies; are the sources whence M. Broussais derives his means of demonstrating the identical nature, and constant seat, of the affections designated by authors by the name of *idiopathic fever*. It remains for us to examine the causes which give to the different shades of *gastro-enteritis* those varieties of character that have induced physicians to separate them from each other.

When the exciting cause of the malady has acted with considerable energy, "we then observe redness and dryness of the tongue, thirst, desire for acids, repugnance to stimulating ingesta, depression of mind, uneasiness about the epigastrium, pain in the abdominal cavity, burning heat of the skin; in a word, all the signs which constitute what has been termed *gastric fever*. If the subject is predisposed to copious bilious secretion, or his bowels are loaded with fæcal matter, the symptoms called gastric embarrassment, either bilious or fæcal, are added to the preceding; and, amongst authors, some say that *bilious fever*, others that gastric embarrassment, are complicated with *gastric fever*." If the patient be predisposed to mucous secretion,—that is to say, of the constitution termed *pituitous;* and bronchial or vesical catarrh is combined with the principal affection; the disorder is then termed *mucous fever*. It differs from the former only from casual circumstances of temperament, or atmospheric influence, having caused a predominance of mucous secretion not only from the gastric organs, but also from the other mucous membranes. In this variety of the disease, the follicles of the mucous membranes appear to be the principal seat of the irritation; and, when it has not terminated favourably within the first twenty days, spots of ulceration, more or less numerous and extensive, are found, particularly about the termination of the small intestine. In some cases there is such a tendency to these erosions, that they appear after the lapse of a much shorter period.

We have shown, in a former article, in what manner the adynamic state of the animal economy was produced: according to M. Broussais, gastric phlegmasiæ, which have not been arrested in their commencement, induce, after a certain length of time, the prostration of strength, the dark colour of the tongue, the fœtid excretions; in a word, all the circumstances which constitute the state termed *adynamic* or *malignant fever*. This state is also the ordinary termination of inflammation of the other larger viscera; such as pneumonia, pleurisy, peritonitis, &c. The fœtid state of the excretions is only a sign of direct debility of the vital powers: it frequently coincides with the most intense inflammatory state, as is seen in angina; the pus of the phlegmon putrefies more readily in proportion as the inflammation is more severe; and, after death, the body passes into a state of decomposition with a degree of rapidity in a direct ratio with the violence of the febrile disturbance that the patient has endured. It appears then to be demonstrated, that the most alarming symptoms which have served to characterize *adynamic* or *malignant fevers* are only the sympathetic results of intense inflammation of the alimentary canal.

It is absolutely the same with respect to the *nervous* symptoms that form the principal traits in the history of *ataxic* or *nervous fever*. M. Broussais observes, that the various phenomena which constitute the state of ataxia may be the results of the influence which the inflamed organs exert on the central parts of the nervous system.

If we analyse the symptoms of the *yellow fever*, *typhus*, and the *plague*, we shall see that, in the first of these diseases, the yellow colour depends solely on the violent irritation of the duodenum, which is propagated to the secretory organ of the bile; all the other symptoms of that fever are those of inflammation of the stomach and small intestines. The researches of Pugnet, Tommasini, Dubreuil, and many others, leave no doubt respecting the correctness of this determination respecting the seat of the disease. We ought, according to M. Broussais,—and the greater number of judicious physicians adopt this opinion;—we ought to apply the term *typhus* exclusively to a disease produced by putrid miasmata, or transmitted by contagion. What some authors have termed *sporadic typhus* is nothing else than gastro-enteritis accompanied with prostration of strength, and the phenomena of irregular action of the nervous system. If we study attentively the effects of deleterious miasmata on the animal economy, we observe—1°, that they may produce even immediate death, by the powerful influence they exert on the nervous system; 2°, that, when the individual is strong and vigorous, there ensues, after the lapse of a greater or less period of time, a febrile state which is owing to irritation of the most sensible of the organs chiefly formed by the sanguineous system: that is, inflammation of the mucous membranes of the stomach and lungs. The nervous system is always severely affected; and thus prostration of strength, and various irregular phenomena, announce its derangement. "The brain, however," says M. Broussais, "is only primarily affected when the effects of certain circumstances have made the action of the exciting cause predominate in that organ; such as moral affections, nostalgia, excessive heat, &c.; but it always suffers much from sympathy, and sometimes to such a degree that the irritation of it passes to actual inflammation, which becomes as violent as if it were primitive." Thus, on the closest analysis, according to M. Broussais, "the varieties of febrile typhus are gastro-enterites ordinarily complicated with pulmonary catarrh; these two phlegmasiæ are the result of the influence of a real poison on the animal economy."

The *plague* presents several points analogous with typhus: the ancients, indeed, for a long time confounded these two affections, and designated, by the appellation λοιμὸς, or *pestis*, all contagious diseases productive of great mortality. The causes and symptoms of the malady, and the appearances observed in the bodies of those who have fallen victims to it, show that the digestive canal is the seat of the inflammation which constitutes the disease. If petechiæ, carbuncles, and inflammatory tumors termed *buboes*, manifest themselves, they are appearances which, as in typhus, are merely the results of sympathetic irritation of the skin and cellular tissue; the development of which irritation is favoured by heat of climate.

Such are the fundamental propositions of the doctrine of M. Broussais respecting inflammation of the gastro-intestinal portion of the alimentary canal. The space within which this subject must be confined will not permit us to relate all the arguments, and physiological and pathological observations, which are adduced as proofs of this important part of his system: we believe, however, that sufficient has been said to enable our readers to embrace the order and connexion of his ideas, and to enable them to supply, by their own reflections, what we may have here omitted to adduce.

From what has been said respecting the causes of *idiopathic fevers*, as they are frequently termed, it is evident that the treatment of them should be founded on the two following indications: —1°, to diminish the irritation in the viscera whence they derive their origin; 2°, to re-establish, by means of counter-irritants, the equilibrium of the vital actions.

The morbid irritation of the gastric system should always be combated by spare diet, cool and acidulated drinks, general blood-letting, if the subject be moderately strong, and in other cases local bleeding by leeches to the abdomen, and the other means usually termed antiphlogistic. These measures should be continued with perseverance, and almost constantly; when the disease has been attacked at its commencement, and the patient has not been subjected to the action of causes which have for a long time over-excited the digestive organs, they are sufficient for the removal of the disease, and effect convalescence in a few days. The success that M. Broussais daily obtains from this conduct; the opposite consequences he has frequently occasion to witness from the indocility of patients; the difference of the results from this practice from those derived from the conduct he had formerly pursued; the authority of the practical observations of Sydenham, Pringle, Huxham, and the most judicious physicians of all ages,—are the motives which induce him to recommend the use of antiphlogistic measures in the diseases we have just considered.

Emetics, so frequently employed in slight gastric affections, he considers a dangerous species of remedy: it is this medicine which, by exasperating the irritation of the stomach, so often causes the slightest affections to pass rapidly to the most violent forms of disease. But the different names that have been given to all those varieties; the vague opinions held respecting their seat, their cause, and the mode of action of the medicine; has prevented the greater number of physicians from appreciating the too-frequently pernicious effects of emetics in these disorders.

The above is the general method of treatment adopted by M. Broussais; various other additional measures are to be adopted, according to the peculiarity of circumstances in different cases. "When gastric irritation is accompanied with excess of bilious secretion, evacuants of the primæ viæ will be proper; but it is important that the period for their exhibition should be judiciously chosen. If the irritation be violent, the use of them should be deferred; and frequently the other measures are sufficient for its removal. Should the symptoms from bilious or fæcal irritation continue, the moment of remission procured by the antiphlogistic measures should be seized for the procurance of evacuations, and the use of sedatives then reverted to. When increased mucous secretion and catarrhal affections are complicated with gastric irritation, the cure will not be so quickly effected, but the treatment is always to be founded on the same basis. If catarrhal irritation persist after the subsidence of vascular excitation, the use of a nutritive diet may be commenced, appropriate to the necessity of the case and the habit of the patient: slight sudorifics, tonics, and even astringents, may be employed, provided that care be taken to proportionate the doses and quality of them to the degree of excitability of the gastric organs."

When muscular debility, contraction of the pulse, torpor of the intellectual functions, &c. announce that the gastro-intestinal irritation has arrived at its highest degree of intensity, is it by placing stimulants in contact with the inflamed organs that we may rationally hope to prevent disorganization and death? Without doubt, we should not in these cases insist on sanguineous evacuation, as in the early stages of the disease: sedatives internally, counter-irritants,—such as, sinapisms to the limbs, vesicatories, &c.—will then be most proper.

"In cases where the febrile state presents itself with the phenomena of nervous disorder, either muscular, sensitive, or organic,—such as convulsions, paralysis, increased susceptability of sensual impressions, exaltation and aberration of the moral faculties, irregular congestion in the viscera, variation in the development of heat, &c."—the *suffering organ*, which is productive of these symptoms, should always engage the particular attention of the practitioner. The fundamental indication is to calm its irritation; then, if the brain appear to be much excited, revulsive measures should be employed against this complication: one of the most proper is the application of ice or cold affusions to the head, whilst the inferior extremities are plunged into a warm-bath. The congestions which take place in other organs may be treated by analogous measures; but the digestive system, the seat of the inflammation productive of them, should never be exposed to additional irritation.

These principles should serve as a basis for the conduct of the physician in the treatment of typhus. After having detailed the terrible accidents which, according to authors, signalize this disease, and the frightful mortality that most frequently accompanies it, "it has only occurred to me," says M. Broussais, "to find similar maladies in the southern part of the Peninsula, where I passed five years, as chief physician of a division of the army; but neither myself nor my colleagues have met with real typhus, except in a temporary manner, under circumstances of forced marches, great want of provisions, &c. We have constantly found it disappear after a short time, when it was possible for us to put in execution salutary measures. By taking care to moderate re-action by the use of acids, &c.; joining with them revulsive measures when the head was affected; and not forcing a patient, whose tongue was black and covered with a hard crust, and who suffered ardent thirst, to swallow wine, cinchona, and camphor; we had to deplore the loss of but a small number of brave fellows: and, if dissection of those who died caused some regret, it was always for not having been able, by sufficiently prompt attention, to arrest the progress of the inflammation that had disorganized the viscera."

*(To be continued.)*

---

***Arthrocacology; or a Treatise on Luxations of the Joints dependant on internal Causes; and on the Efficacy, Modes of Action, and Application, of the Actual Cautery, in the Treatment of those Diseases.*** By J. N. Rust, Professor of Medicine and Surgery to the Royal Military Academy at Berlin, &c.—4to. pp. 195. Vienna, 1817.

*(Continued from page 184.)*

In support of the opinions he has advanced respecting the original seat of the disease treated of in this work, Professor Rust observes, that the malady first shows itself by pain and swelling about the extremities of the long bones; which, when the disease affects the shoulder or any of the more superficial joints, are very evident both to the sight and touch; whilst the socket of the joint remains in the healthy state. The patient also complains of tenderness of those parts on their being handled. The gradual increase of the swelling, and the dislocation of the condyle from the articulating surface in consequence of its enlargement, may also be traced in those joints which are but thinly covered by integuments: this, however, cannot easily be detected in the hip-joint, in consequence of the voluminous muscles by which it is enveloped. But, in the latter case, the peculiar kind of pain that is experienced about the upper part of the thigh will point out the seat of the affection. The observations of Camper, Ford, Albers, and some other writers, the author remarks, do not accord with this statement; and he himself has witnessed some instances where the disease appeared without the pain and swelling above described. But, a careful investigation of these cases has shown that the disease had been accompanied with those phenomena in the first instance, but that a longer or shorter period had elapsed between the original affection and the occurrence of the lameness, during which the disease was not clearly apparent; and thence dislocation of the bone took place without having been apparently preceded by its real cause. He continues to observe, that Fabricius Hildanus,* Desault, Jager,† and many other writers, have observed that the hip-joint disease commenced with pain about the upper end of the femur; and that Asclepiades also mentions pain in the thigh as the original symptom of the disease that produces the dislocation of the hip-joint. The analogy of this affection with those arising from the scrofulous, rachitic, and other morbid diatheses, which are known to affect the extremities of the long bones, tends also to support the opinion here advanced.

But it is from examination after death that the author deduces the most powerful evidence in support of his theory. There are many cases, he observes, in which the cotyloid cavity, as well as the head of the femur, is found destroyed by caries, and in which it may be impossible to trace the original seat of the disease; but there are others, on the contrary, which prove that it begins in the extremity of the femur, and thence extends to the joint. In a girl, who died at twelve years of age from scrofulous pulmonary consumption, he found the cavity of the hip joint in the natural state, whilst the head of the femur was partially removed from the socket, and the extremity of it, for some distance downwards, thin, and nearly hollow from caries. In a man who died in the last stage of the hip-joint disease, the head of the femur was completely dislocated, and a joint formed in its new situation; the extremity, to a little distance below the great trochanter, was nearly destroyed by caries, but its articulating surface was covered by healthy cartilage; and there was hardly any diseased appearance in the acetabulum. In another case, where the patient had been unable to move the limb for three months, from the severity of the pain induced by the attempt, the head of the femur was found covered with healthy cartilage, though the round ligament was destroyed by ulceration, and the upper end of the thigh-bone much corroded by caries. There was no diseased alteration in the acetabulum, except a little roughness where the round ligament

---

* *Cent.* vi. *Obs.* xci.

† *Funfzig Chirurgisch-praktische Cautelen für Angehende Wundärzte.* S. 147, bis. 169. Frankfurt, 1788.

was attached. Analogous appearances were also observed in other cases that affected the shoulder-joint. Professor Rust says he has inspected many anatomical preparations, that show the disease to have had the origin which he has described; and he refers to observations of other pathologists, as Paletta, Ford, Hoffmann, and Albers, for facts which also tend to substantiate the correctness of his opinions.

Preparations are, however, to be met with amongst collections of morbid anatomy, in which the acetabulum is found to have been affected with caries, without being accompanied with any alteration of the head of the femur; but these, Professor Rust observes, do not prove any thing against his theory, since precise histories of the cases are always wanting; and he himself possesses one which elucidates the origin of all the rest: in this case, the caries of the acetabulum was the consequence of a collection of matter arising from diseased lumbar vertebræ, which, by resting on the interior of the pelvis, had caused the destruction of the bones, and thence the disease extended into the cavity of the hip-joint; and he continues to remark, that, notwithstanding the numerous opportunities he has had for observation, he never saw a case where the morbid change commenced in the acetabulum, and thence extended to the head of the femur.

Considering the disease in this point of view, Professor Rust thinks the terms *coxalgia*, *spontaneous* or *consecutive luxation*, &c. which have usually been applied to it, to be improper; and that *arthrocacia*, which the ancients applied to caries of the articular ends of the bones, without any direct relation to spontaneous luxation, would be more appropriate as a common appellation. The affection should then be termed *coxarthrocacia*, in the groin; *omarthrocacia*, in the shoulder; *olecranarthrocacia*, in the elbow; *gonarthrocacia*, in the knee; *spondylarthrocacia*, in the vertebræ, &c.

The author next enters into the consideration of the remote causes of the disease; but little that is novel or satisfactory is advanced on this point; we shall, therefore, pass over this part of the work to that which treats of its diagnosis. We must here observe, that our limits will permit us to give only a very imperfect sketch of the clear and energetic picture drawn by the author. This part of the work is written with extreme care and methodic precision. Every symptom and sign of importance tending to mark the nature and progress of the disease is noticed in a manner that evinces great acuteness of observation and judicious discrimination.

We shall first notice the phenomena of the disease that are common to it under every variety of situation, and then describe the peculiarities in its character which appear in the *coxarthrocacia*, the most frequent and severe form of this affection. The progress of the malady is divided by the author into four distinct periods.

The first stage of the disease is characterized by erratic pains about the extremity of the bone, and sometimes by a slight degree of weakness of the limb and restraint of its natural motions, before any evident change in the form of the joint can be remarked. At this period the disease consists in an inflammation of the medullary membrane of the head of the bone.

In the second stage, the central portion of the bone begins to be affected with caries, and the form of the parts about the joint is evidently changed. The head of the bone becomes enlarged and softened; it recedes from the other articulating surface of the joint, and an increase in the length of the limb necessarily ensues.

The caries continues to make a more or less rapid progress; the head of the bone is gradually destroyed, the limb is drawn into different directions according to the influence of the surrounding muscles, &c. and the limb becomes shortened: this constitutes the third stage.

The fourth period commences when the disease, extending itself to the soft parts surrounding the joint, produces profuse suppuration of them. Hectic fever also now takes place, and the malady soon terminates in death, unless arrested without delay.

In the *coxarthrocacia*, the first stage of the disease is announced by a deep-seated peculiar kind of pain in the upper part of the thigh, which disappears and returns from time to time; some degree of stiffness is also felt in the groin, accompanied with a slight lameness and a sensation of weakness in the limb, particularly on using a little exertion. On the approach of the second stage, which sometimes occurs in a few months, at others not until after the lapse of some years, the affected limb is found to be somewhat longer than the sound one; the great trochanter is lower, and projects outward more than ordinarily; the nates are flattened, and the natural depression in them behind the trochanter is increased in depth; the whole limb is also somewhat wasted. A severe and almost insupportable pain is now felt in the knee, and some degree of swelling of that part is sometimes evident. After the lapse of a longer or shorter period of time, the head of the bone gradually slips from the socket, though sometimes this occurs suddenly; and the disease has arrived at the third stage. The affected limb is now shorter than the other; the thigh is more bent than formerly; the great trochanter is turned more inwardly, and the thigh has assumed more of a conical figure. It is not uncommon to find the symptoms somewhat relieved at this period, but this is only of short duration: the pain about the knee returns with all its former violence; hectic fever comes on, or increases in severity; tumefaction about the articulation and the upper part of the thigh becomes evident, and a fluctuation in those parts may be discovered. The fourth stage now commences. The skin about the joint gives way, and a large quantity of serous and purulent matter escapes, which is followed by some alleviation of the symptoms. The pus soon acquires an ichorous character; the strength of the patient decays; his sufferings increase; he becomes affected with colliquative sweats and diarrhœa, and death comes to end the long and distressing scene. But, some few instances have occurred where, even in this state, the disease has been cured, leaving only an anchylosis of the joint and a shortening of the limb.

After having noticed the occasional deviations of the disease from the general course above described, and treated of each of those variations with all the care and precision so interesting a subject deserves, Professor Rust then considers the different affections that may possibly be confounded with it.

Although a careful investigation of the symptoms will render evident the nature of this malady, yet, as it may tend to facilitate the diagnosis at an important period, we shall adduce the comparison of the author between this and the affections that bear some resemblance to it in its early stages: the chief of which are, the *natural lameness of children from mal-formation of the bones of the pelvis*, described by PALETTA;* the *nervous sciatica* of COTUNNI;† *psoas abscess;* and *separation of the head of the femur from its neck, from internal causes.*

In the natural lameness of children, the limb differs in length from the sound one in the first instance; in the hip-joint disease, on the contrary, the length of the limb does not, in the first stage, vary from that in the natural state; and it may be useful to remark, that the shortening of it, which subsequently occurs, is always preceded by an increase of its length. In the former disease, the disproportion of the length of the affected limb will also frequently disappear when the child is placed in the horizontal posture, and the limb may be extended without much pain being induced: in the coxarthrocacia, on the other hand, the difference in the length of the limb is apparent whether the patient be in the erect or horizontal position, and it cannot be extended without causing severe pain. The natural lameness is not accompanied with any alteration in the form of the nates, or they are merely a little flattened, and are not diseased with respect to structure: in the coxarthrocacia, we find the nates much flattened during the lengthened state of the limb, and more prominent and firm than natural when it has become shortened. In some other affections that are accompanied with shortening of the limb, the nates are thinner and flatter than in the coxarthrocacia. The motions of the joint are tolerably free, and effected without pain, in the disease to which we first alluded; but, in the latter, the motion of the limb is restrained, and accompanied with pain, when it has made a little progress. In the natural mal-formation of the joint, the patient, in walking, places the sole of the foot on a level with the ground; whilst, in the coxarthrocacia, he is observed to tread on the toes only of the affected limb.

The *nervous sciatica,* so accurately described by Cotunni,† cannot be confounded with the coxarthrocacia, if the following diagnostic signs be attended to:—

---

* *Adversaria Chirurgica*, i. 4.

† *Commentatio de Ischiade Nervosa.* Viennæ, 1770.

In the nervous sciatica, the pain is experienced in the hip, chiefly behind the great trochanter, and extends from the os sacrum, along the course of the sciatic nerve, downwards as far as the outer part of the foot: in the coxarthrocacia, the pain is not unfrequently wanting in the first stage of the disease, (a slight lameness and stiffness of the joint being alone experienced,) and, when it is present, it is generally referred to the upper and anterior part of the thigh, never extending in the course of the sciatic nerve, and frequently being confined to the region of the knee-joint. Sometimes the anterior femoral nerve is affected in the same manner as the sciatic; this may be recognized by the pain being increased by pressure in the course of the nerve, particularly where it issues from the pelvis under the fallopian ligament. The patient afflicted with sciatica becomes crippled, and cannot go about; but, in the coxarthrocacia, although he is affected with some degree of lameness, he can, in the first stage of the malady, move from one place to another with at least a moderate degree of activity. No alteration in the state of the soft parts in the region of the great trochanter can be observed in the sciatica; but this is not the case, as before stated, in the hip-joint disease. No stiffness of the joint, nor variation in the length of the limb, take place in the sciatica; or, if the latter does occur, it will be readily discovered to arise solely from obliquity of the pelvis. The pain in this complaint is also different from that in the hip-joint disease; it is not so deep-seated, and is frequently accompanied with spasmodic contractions of the muscles of the limb.

The affection we are considering may, however, be more readily confounded with *psoas abscess*, as those maladies have many symptoms in common. The flattened appearance of the nates in their first stages, and the subsequent tumefaction of those parts; the formation of abscesses, showing themselves about the upper part of the thigh; the lameness; the pain on extending the limb; and the particular uneasiness in the upright posture; are equally experienced in both diseases. The diagnosis may, however, be formed by an attention to the following circumstances:—

In inflammation and suppuration of the muscles about the loins, the patient complains of pain in the region of the affected parts, particularly in assuming the upright position of the body, or on extension of the lower extremities. We need not point out the dissimilarity between these symptoms and those arising from the hip-joint disease. The former complaint also runs its whole course without producing any uneasiness or change of structure in the region of the great trochanter, and the foot of the affected side cannot be turned outward without increasing the pain; whilst, in the hip-joint disease, the foot is in general turned more outwardly than in the natural state. In the latter stages of the disease, when abscess has formed, it will be found, in the affection of the psoas muscles, that a deep inspiration, coughing, hollowing, or the erect position, will be accompanied with a fluctuating swelling about the nates or the anterior part of the thigh; or, if the abscess has burst externally or been artificially opened, an evacuation of matter from it will take place: these circumstances are not witnessed in the coxarthrocacia.

ROLFINCK,* PERÆUS,† OVERKAMP,‡ DIEMERBROECK,§ SCHRADER,|| MOEGLING,¶ WEDEL,** and several other authors, speak of a disease arising from the spontaneous separation of the head from the neck of the femur, which produces symptoms much resembling those of the coxarthrocacia; but Professor Rust says he has never himself witnessed such an occurrence; and the description given of it by the above authors is so various, that he, with ALBERS and PALETTA, doubts of the reality of its existence. He thinks that some of them have mistaken a fracture of the neck of the femur, or the latter stages of coxarthrocacia, for the affection above mentioned. Should it, however, occur, the symptoms must, from what has already been stated, be so easily distinguishable from those of coxarthrocacia, as not to merit particular consideration in this place.

We pass over the history of *arthrocacia* as it appears in the other joints, because the account we have already given of it will be sufficient to lead to the knowledge of the disease in all its various situations; and, as we before stated, it is not our intention to give a complete analysis of the work of Dr. Rust, but merely such parts of it as are either particularly interesting, or tend to elucidate his particular theory of the nature of the disease. We must not, however, neglect to notice this morbid affection as it appears in the articulation of the atlas with the dentata vertebra, as it has not, until very lately, been treated of by any modern writer, and it is productive of some peculiar symptoms by which it may be distinguished in its early stages, when remedial measures are alone of any considerable utility. Professor Rust has witnessed thirteen cases of this species of the disease.

The patient at first experiences pain in the back part of the neck, which is more severe in the night, during wet weather, when he attempts to swallow very small portions of food, or on making a deep inspiration. After a certain length of time, the pain becomes more violent, when the head is inclined to one side. Powerful pressure with the finger about the union of the head with the neck is then found to excite a very disagreeable sensation, which points out the true nature of the disease. If it be now neglected, the difficulty of swallowing and respiration continue to increase, and the pain, which is concentrated about the occiput,

---

* BONET *Analect. Pract.* lib. iv. p. 6. *Observ.* 2.

† Lib. xvii. cap. 22.

‡ *Chirurgia*, p. 894.

§ *Anatom.* lib. ix. cap. 16, 19.

|| *Dissert. in Hippocrat. Aphorism*, sect. iv. 43; *in Illust. Aph.* 59.

¶ *Ephem. Nat. Curios.* cent. v. p. 220.

** *Exercit. Pathol. Therapeut.* p. 126.

becomes almost insupportable on the least motion of the head. The head now falls on one shoulder, and usually on the right one, because the disease most frequently affects the left side of the vertebræ in a particular manner. About this stage of the malady, the state of the patient becomes somewhat alleviated, the motions of the head are more free, and it even recovers in some degree its erect position. But this state is of but short duration. The pain in swallowing, speaking, and breathing, recurs; the head becomes inclined a little backward, or to the side opposite to its former position. The patient cannot obtain ease in any posture; and he can neither rise nor lie down without supporting his head with both hands. Paralysis of the upper extremities, loss of the voice, and hectic fever, then ensue; and a termination is soon put to the patient's existence. Nothing remarkable is generally observable externally: Professor Rust, in one instance only, witnessed a tumor in the situation of the diseased bones, which burst outwardly, and was followed by a fistulous ulcer. The expression of the countenance, in consequence of the pain experienced, is so peculiar, and in some degree characteristic of the disease, that the author has inserted in this work the portrait of a patient affected with it in its latter stage. We hardly ever witnessed so distressing a representation of the human visage: the fixed teeth, curled lips, obliquity of the eyes, contracted brow, rugous forehead, erect hair, and the sharp lines formed by the muscles in consequence of the state of emaciation, give to it an expression that the excellent artist (J. Giller), who executed it, has well displayed, but which cannot be effected by the powers of any language.

We shall pass rapidly over that part of the work devoted to the consideration of the treatment of arthrocacia, since the general measures advised by the author do not differ, except in the use of the actual cautery, from those so well pointed out by our own writers, especially by Mr. Brodie in his recent work on the Diseases of the Joints.

Professor Rust first treats of the general measures that are usually employed, amongst which he assigns the first place to blood-letting. The free application of leeches has, indeed, been sufficient to arrest the progress of the disease, when it has been of but short duration, or arisen from external violence, or when it appears to have been essentially connected with a plethoric state of the general system. He next considers the various internal measures that have been advised by different authors; as antimonials, sulphurous preparations, the extractum pampinorum vitis (recommended by F. Hen. Peter Frank), mercurials, the woods usually termed antisyphilitic, tepid baths, nitric acid, muriate of barytes, opium, aconite, digitalis, &c.; but we need not dwell on this part of the subject, as it is on the local treatment that the chief and only confident dependence should be placed.

In the first stage of the disease, the author has found leeches repeatedly applied to the surrounding parts, of great utility; after which, stimulating sinapysms, vesicatories, volatile liniments, a particular preparation with which we are not acquainted (AUTENRIETH's*chen Brechweinsteinsalbe)*, warm plasters of mastic and gum ammoniacum, &c.; but the author has found no measures so effectual at this period as mercurial frictions. After having relieved the severity of the pain by blood-letting, leeches, and warm baths, he advises the joint to be rubbed with mercurial ointment until the disease subsides, or the occurrence of salivation requires the discontinuance of the remedy. When the complaint has arrived at the second stage, a more powerful counter-irritation is necessary. The establishment of issues in the neighbourhood of the joint, Dr. Rust remarks, has been long considered as sufficient for this purpose; but numerous observations and long experience have proved to him that they but rarely produce the desired effect, and that they even accelerate the progress of the disease when exarticulation has taken place. He considers the actual cautery as entitled to a decided and eminent preference. But we need not dwell on that part of the work which relates to the use of this remedy, for we fear that popular prejudice would prevent its introduction into general practice by English surgeons, should they even be convinced, by the unanimous voice of the most eminent surgeons of the whole continent, that they have neglected the use of a powerful remedy for which there is no efficient substitute. We may, however, remark, that the same principles that are inculcated by our writers for the use of the caustic, are also applicable to the actual cautery. We cannot pass over this section of the present work without observing, that it contains such a full, explicit, and methodic account of all the different circumstances to be attended to in the use of the remedy, under every variety and form of the disease, and such numerous references to rich sources for information in other writers, as must render it a highly valuable possession to the practical surgeon.

When the disease has arrived at the third or fourth stage, a perfect cure can no longer be expected; we must then only endeavour to arrest the progress of the malady, to limit its deleterious influence on other parts, and favour, as much as possible, the efforts of nature to cicatrize the ulcers. The cautery is still, however, the best remedy; but, if the collection of matter is large, it will be necessary to evacuate it previously to the use of that application. The author here adduces some very judicious reflections on the treatment of abscesses in general, and on those connected with the joints in particular. He blames the custom of evacuating these collections by a small opening, under the pretence that the contact of air alters the qualities of the pus. It is not, he says, the contact of air, but the diseased state of the parts, that is the cause of the bad qualities of the pus; for, whether it be large or small, the opening will always give passage to air; and, when it is small, there is the disadvantage of a pouch being formed, in which the matter becomes collected, and air is confined and altered without the possibility of its renewal. To avoid the inconveniences that have been gratuitously attributed to too large incisions, it is necessary to imitate the natural operations, in which an abscess never opens until the integuments covering it have acquired a state of inflammation proper to favour the prompt obliteration of the opening after the pus is evacuated. Professor Rust, therefore, advises, in the cases of abscess from collection of matter now under consideration, that a large incision should be made, so as to give immediate issue to the contained fluid; but that this should not be done until the skin and adjacent parts have been forcibly irritated by cauterization or other measures, so as to excite their vitality, and place them in a condition the most favourable to prompt adhesion of the divided parts.

We shall here terminate our analysis of this work, having somewhat exceeded our professed object, which was to give our readers a general knowledge of the most interesting of the novel opinions of the author, rather than to attempt to furnish a sufficient idea of the multitude of interesting observations, and the useful practical information which it contains.

---

*An Essay on the Consequences of the more severe States of Child-Birth;* by J. E. Fodéré, M.D. Professor to the Faculty of Medicine at Strasbourg.

"Notwithstanding the contests which still divide the accoucheurs of the different nations of Europe, it is certain that the department of the medical art which relates to the state of child-birth has not yet made any considerable progress. Every point of the head of the fœtus has been placed in opposition with those of the canal through which it must pass; the relative advantages and inconveniences of turning, and the application of the forceps, have been weighed; that instrument has been carried with a bold hand within the limits of the abdominal cavity, and it has recently received those modifications of its form, relative to the different degrees of inclination of the pelvis, which had been anticipated by Levret. Nothing, then, it would appear, should henceforth oppose the delivery of a woman by the ordinary means, whenever her conformation does not offer insurmountable obstacles to it. Besides, the means of preventing, to a certain extent, the extremities which have been resorted to under these serious circumstances, have already engaged the attention of the professors of the obstetric art; an object which will form the subject of a future memoir. Well! the woman is delivered, and favourably so, either by the powers of nature alone, or by the aid of vigilant art; yet she still must encounter considerable dangers: this dear object of the solicitude of a father, of a husband,—this mother, so happy in pressing to her breast the infant that had caused her such sufferings,—may probably perish within a few days. This spectacle, of which I have frequently been a witness, and which is so often renewed, even in those great cities that most pride themselves for the possession of men of enlightened talents,* has always excited in my mind the most poignant feelings of distress, has irritated me against the inefficacy of the art, and determined me long since to observe with assiduous attention whatever appeared to presage, or might tend to avert, the occurrence of similar calamities. It is the general results of my labours that I now present to the public, not as a perfect production, but merely as advice to practitioners, to place them on their guard on such occasions.

I shall commence with some physiological considerations respecting the state of pregnancy and child birth, for it is thence I shall deduce all the observations of utility that I may have to propose; I shall treat of the principal maladies to which the woman in the latter state is subject, in consequence of her peculiar situation; I shall expose, in the third place, my sentiments respecting *puerperal fever*, its complications, and its appropriate treatment; I shall discuss, in an independent manner and from practical observations, the opinions that have been advanced respecting this serious affection; and, lastly, I shall endeavour to appreciate, by arguments also deduced from facts, the importance of those disorders commonly termed *milk-fevers.* I have a double object in the publication of this Memoir,—that of conferring some benefit on the most interesting part of human nature, and that of putting a stop to some ridiculous disputes now in agitation: may my labours and arduous efforts not be totally devoid of utility!

A very ancient prejudice, which has its source in the works of Hippocrates, where the uterus, pictured as an animal contained within another animal, is represented as travelling throughout the three cavities of the human body, attributes to that organ, even in the state of vacuity, the greater part of the maladies which afflict the female sex: thence the name *hysterics* given to convulsive affections, to which men are equally subject with women. It is nevertheless certain that the uterus exerts no activity on the general system, except during the time of gestation, and only makes itself an object of perception at the periods of menstruation, and by the organic diseases which are common to it with other parts of the body. But, as soon as a woman has conceived, then, indeed, this mute organ assumes a new life, which modifies it, and produces an influence on the whole existence of the individual.

---

* Of about 20,000 deaths that take place annually in London, there are on an average 230 of women in child-bed: at the *Hospice de la Maternité*, at Paris, there is ordinarily the loss of one woman in twenty-five in that state; (*Compte rendu de l'Administration* in the *Journal de Médicine* of M. Leroux, Juin 1817:) of 717 women delivered at the Civil Hospital at Strasbourg, under the superintendance of M. Lobstein, we find 61 died; and, of 13 cases related of *puerperal peritonitis*, only three recovered; (*Observations d'Accouchemens*, &c. par J. F. Lobstein; pp. 56 and 57; Paris, 1817.) It must, then, be readily acknowledged, that, for a natural function, child-birth is too often a fatal one.

The dark circle round the eyes, the languishing look, the alteration of the traits and colour of the countenance, deranged appetite, nausea, vomiting, and other general changes which follow conception, indicate that the different functions are going to become subordinate to that which appertains to the preservation of the species. The uterus acquires, by conception, two active properties which did not previously exist in it, and which I willingly term, with M. Deneux, *animal sensibility*, and *sensible organic contractility*.* By the first, that organ enters within the dominion of pain, which the woman begins to experience as soon as the fœtus exerts considerable motion, and which will be still more acute at the period when its contractions are to effect its expulsion. By the second, the uterine tissue acquires the faculty of becoming rapidly very firm, then of spontaneously relaxing itself; and this alternately during a given time. But, besides this, it becomes susceptible, from the commencement of gestation, of a dilatation that it would not have been supposed capable to undergo, not a mere elongation of its tissue, as in diseases, but an active dilatation, —that is to say, which depends on augmented nutrition, on an addition of molecules, which permit that organ considerably to extend its limits without suffering a diminution of the thickness of its parieties. There results from this dwelling that nature forms for the new being from almost all the species of structure which constitute the human body, and from the increase of this being and its appendages, that the pelvis of the mother becomes a centre of fluxion, which draws all towards itself, and to which the greater proportion of the vital powers tend, very often to the injury of the rest of the economy. The same circumstances occur in the females of other animals as in women; but the latter, possessing a nervous system infinitely more extensive and more developed, is also much more affected and modified by the great change which then takes place.

Let us consider the circumstances which should particularly engage our attention, as those which constitute dispositions to disease during the period of child-birth. We may enumerate seven of particular importance:—1. The shock given to the bones of the pelvis. We believe, with many authors, from facts that have occurred to our observation, that, in every instance of labour, the head of the infant, engaged in the pelvis and propelled by the action of the expulsive powers, exerts on the sides of this cavity an effort that has a tendency to separate the different bones composing it, and which necessarily produces painful distension of the ligaments and cartilages, which obliges the woman to remain in a state of repose during a greater or less length of time.—2. The increased sensibility of the uterus and its appendages, and the disposition of those parts to inflammation. It is almost useless to

* We adopt the word *contractility*, as there existed no word in our language which conveys the same precise idea: *contractibility* has an absolutely different meaning.—Edit.

insist on this point. Although we have seen women, after a clandestine delivery, return immediately to their ordinary occupations, there is no one who will not acknowledge that the labour of child-birth must produce a painful state of the uterus and all the organs contained in the pelvis, and even of the abdominal muscles which have participated with the expulsive efforts; that the pressure of the fœtus on the soft parts will produce a state of them analogous to that from contusion in general; that this state of painful sensation will be greater in proportion to the frequency of the repetition of the uterine contractions, the largeness of the head of the fœtus, the length of time which the head may have rested in the pelvis, and the degree of violence with which it may have been wedged therein: it will also be more severe when the fœtus has been in a very languid state, or dead; and, lastly, in the first instance of parturition.—3. The degorgement of the uterus, which is effected, either by the contraction of the tissue of this viscus, producing the lochiæ, which is the ordinary result; or by the absorption of the fluids contained in its vessels, without the appearance of lochiæ, or at least a flow of them during one or two days only, which we have witnessed without inconvenience: the latter is a rare occurrence.—4. A disposition to hæmorrhage; which depends on an effort similar to those, as Stahl observed, which persons suffer that have been accustomed to sanguineous evacuations, and then, from some accidental cause, have them suppressed for a considerable length of time, and to which women of a sensible habit of body are very subject; or from a want of contraction, irritability, or contractility of tissue, a default that has been termed a state of inertia of the uterus.—5. The results of the pressure and distension that has been long exerted on the circumjacent parts during the development of pregnancy, which pressure and distension, independently of the affections relative to sensibility, have necessarily deranged digestion, caused the accumulation of fæcal matter in the intestines, and induced a painful state of those organs as well as of the peritoneum, and have given rise to a stagnation of matters ordinarily excreted with more or less promptitude.—6. The general disturbance of sensibility which the endurance of the pains of labour has produced. Although women may be somewhat fortified by example, they cannot but consider that parturition exposes them to much danger. Many, during the whole period of pregnancy, are occupied in the contemplation of the hazard they have to undergo, and, in the midst of the sufferings of child-birth, feel certain that it will prove fatal to them. Besides, although we, in our books, very quietly give the name of a function to parturition, it is not less true that it is a very painful and difficult action, even to a person of ordinary sensibility. It may be compared in many instances, as several respectable authors have already done, with the commotions arising from severe mental impressions, extensive wounds received while influenced by violent passions, or the more severe surgical operations which are submitted to under a full conviction of the dangers about to be encountered.—7. The lymphatic diathesis, which is established towards the termination of pregnancy, and continues after the birth of the infant, and the influence of which is particularly directed to the breasts by means of a revolution in the animal economy very troublesome to many women, and which almost always excites an attack of fever, the occasion of the development of severe diseases peculiar to this state.

The concourse of those severe circumstances places the woman in child-bed in a particular state, different from that of any other situation. Daily observation evinces, at least in practice in cities, that sensibility is then excited to its highest degree of exaltation, and that a thousand impressions, formerly indifferent, or followed by only moderate sensation, may then produce the most serious effects; that a woman, previously mild and reasonable, becomes then irascible and outrageous on the most trifling occasions; that the frustration of the desires of the mother relative to the sex of the infant has caused syncope, convulsions, and other alarming symptoms; that improper diet will readily produce gastric fever; that the impression of cold air often causes catarrhal affections of the whole of the mucous membranes; that if, during pregnancy, the progress of many chronic diseases with which the woman may have been affected, has been suspended, child-birth furnishes an occasion for their more rapid development; and that, above all, it is seldom that women in this state escape epidemic maladies,—so that their situation is a favourable condition for the reception of the febrile element of the wards of an hospital, even when these causes are not sufficient to affect persons in general, and it is from this that hospitals are so frequently fatal to lying-in women. In some years we observe epidemic diseases that affect them exclusively. Acute disorders display in them symptoms of severity that are not common to the respective affections, and almost all fevers assume a degree of intensity, an irregularity in their course, and a nervous and typhous character, which every instant disconcert the practitioner, and prevent any calculation respecting crisis or critical days. Now, should not a state that resembles no other, which has its peculiar phenomena, which engenders maladies that appear in no other condition of life, be designated by a particular name? I think it has been thus considered by some really observant physicians, who have termed it the puerperal state, or *puerperality*; a denomination which should be preserved, and which I shall employ in this Memoir.

As I must here confine myself to the principal dangers present during puerperality, I shall refrain from the deduction of a detail of the consequences which the circumstances above enumerated present for the cautions that should be taken in the management of women in child-bed. My intention is to make some remarks, in the first instance, on the inclination to hæmorrhage, and on that singular disposition to mania which women experience after delivery. I shall afterwards treat more particularly on puerperal fever, and on the explanations that have been given of the nature of that disease. The examination which I shall make of this fever, from a view of its symptoms, its terminations, and the considerations already stated, will serve, I hope, to determine the ideas that should be adopted respecting it, and will explain the cause of the sudden death of so many women in child-bed in the midst of the most favourable expectations. I shall reserve, for the termination of my labour, the circumstances which relate to the seventh circumstance of puerperality.

I have already pointed out the two principal sources of those alarming and often fatal losses of blood, independently of those which are owing to the rupture of a vessel, or to the retention of some portion of the placenta in the uterus; the latter are cases of more rare occurrence, and are more easily remediable. It is not immediately after delivery that this loss is most to be feared; there is at first only a moderate evacuation; but it is some hours after, when a state of calmness has succeeded to the state of commotion that the woman had experienced: it is most frequently even during the first sleep, of which she appears so much in need, and which, nevertheless, we are obliged to interdict when we are in doubt respecting the probability of such an occurrence; at least, if the patient be not extremely exhausted.

The hæmorrhagic efforts, of which I have already spoken, and of which the consideration seems to be generally neglected, appear to me to be as frequent a cause of loss of blood as the inertia of the uterus formerly described. We should always fear them in women of a strong and vigorous habit of body, accustomed to the free use of wine, aromatics, and a luxurious regimen, as well as those who have habitually lost much at their periodical evacuations, and who have a disposition to hæmorrhages. The occurrence of them is imminent, when we witness a universal spasmodic contraction, when the patient complains of shivering and her pulse is accelerated, and when the quantity of blood evacuated is not in proportion with the natural flow of the lochiæ. Sleep favours this species of hæmorrhage, as well as some other disorders, in a very remarkable manner. We know that it is during this state that the menses begin to appear each month in the greater number of women; and I have constantly observed, in those who have never gone the full time of pregnancy, that it was during sleep, and after midnight, that abortion took place in consequence of spontaneous hæmorrhagic efforts.

We must fear the loss of blood from inertia of the uterus, when patients have borne a great number of children; when they have experienced, during gestation, either depressing moral affections, hæmorrhages, or long diseases; when the expulsion of the fœtus and its membranes has been too prompt; and particularly when the uterus does not assume the globular form, and the orifice of that organ remains soft and dilated. Sleep is not less dangerous in this state, because of its sedative and relaxing effect, and the woman has need of the excitation of wakefulness, united to the assistance of art, to induce the necessary contractility of the uterus, without which the life of the patient would flow away with her blood. This is, perhaps, one of the cases where, in adding some degree of power to the natural efforts of parturition, (as Puzos observed,) that we may preserve in the uterus that faculty for contraction that is so necessary a circumstance to the welfare of the patient.

The therapeutics of the first species of hæmorrhage requires the preservation of an ancient custom established for its prevention, and active measures for its suspension when it occurs; that is, we should, to fulfil the first indication, bleed plethoric women when the epoch of labour is about to approach. We should expose her to great danger during the hæmorrhagic effort, if we took the effects for those arising from the want of contractility. I have seen, in such a case, the plug, which an ignorant practitioner had applied, expelled with force by a new flow of blood, red, warm, and concrescible; when two copious blood-lettings from the arm, which I afterwards had practised, in spite of the disapprobation of the attendants, cold-water acidulated with lemon-juice taken as drink, and repose, placed the life of the patient out of danger. I have, on the contrary, employed the plug with success in the second species, when frictions, injections, and affusions of cold water, and acidulated drink, had been of no service. The plug acts here not so much in an express mechanical manner, as by producing constant irritation of an organ that has not yet lost all its sensibility.

It is not only by the loss of blood that these hæmorrhages are dangerous, but also by the fevers of an ill character that they dispose the patient to contract, in consequence of the extreme debility into which they are plunged, and from which they therefore encounter a double risk. I was called, in 1811, to Marseilles, to the wife of a mason, the mother of several children, who was in the ninth month of pregnancy, and had suffered a considerable loss of blood during several days. She was pale, exhausted, and her tongue even was colourless and without warmth. Having, by examination, discovered that the orifice of the uterus was dilated, the placenta separated and lying near the orifice, I sent to request the assistance of an able accoucheur of the city, Dr. Giraud Saint-Rome; who, after having recognized the same circumstances, delivered the woman by turning, without retracting his hand. The hæmorrhage ceased; but it returned after the lapse of a few hours, and showed itself from time to time, notwithstanding our endeavours to prevent it. No determination to the breasts ensued, and, instead of milk fever, all the symptoms of putrid fever supervened. We determined to employ ipecacuanha, according to the method of Doulcet, less as an evacuant than as an excitant of the whole system. The patient took it, at short intervals, during several days. The first occurrence of vomiting caused a cessation of the sanguineous evacuation. The danger continued great until the third day, and the fever did not subside before the fiftieth, at which time the woman was in a state of convalescence. Two years afterwards, during a short stay that I made at Avignon, I was called in consultation on the case of a young woman, of a robust habit of body, and naturally of a violent disposition. She had been delivered ten days, had suffered a considerable loss of blood that the medical attendants had not been able to suppress, and was then attacked with putrid fever. They had followed the method of Doulcet, and also administered purgatives; but the aspect of the patient, the antecedent circumstances, and the appearance of the blood evacuated, announced the existence of hæmorrhagic efforts rather than a state of inertia of the uterus. New measures appeared to arrest the progress of the disease for some time; but I learned, after my departure, that the woman had fallen a victim to the malady: so true it is that the nature of sanguineous effusions should be distinguished, and that it is necessary to stop them without delay by proper treatment, to preserve the patient from the dangerous febrile affection that often follows them in the state of puerperality.

*(To be continued.)*

---

